# Draft Genome Sequence of Environmental Bacterium *Vibrio vulnificus* CladeA-yb158

**DOI:** 10.1128/genomeA.00754-15

**Published:** 2015-07-23

**Authors:** Yael Danin-Poleg, Nili Raz, Francisco J. Roig, Carmen Amaro, Yechezkel Kashi

**Affiliations:** a Faculty of Biotechnology and Food Engineering, Technion-Israel Institute of Technology, Haifa, Israel; b Estructura de Investigación Interdisciplinar en Biotecnología y Biomedicina ERI BioTecMed, Departamento Microbiología y Ecología, University of Valencia, Valencia, Spain

## Abstract

We report the genome sequence of the environmental *Vibrio vulnificus* biotype 1_cladeA. This draft genome of the CladeA-yb158 strain, isolated in Israel, represents this newly emerged clonal group that contains both clinical and environmental strains.

## GENOME ANNOUNCEMENT

*Vibrio vulnificus* is an aquatic bacterium and an important human pathogen (1–4). Strains of *V. vulnificus* are biochemically divided into three biotypes. Biotype 1 is a worldwide distributed pathogen and highly varied (5, 6). Recently, we found a new phylogroup, clade_A, which includes both environmental and clinical isolates and presents biochemical characteristics that differ from those of biotypes 1 and 3 (7, 8). Here, we describe the draft genome sequence of the environmental *V. vulnificus* biotype 1 strain CladeA-yb158, isolated in 2005 from tilapia fish grown at aquaculture ponds in northern Israel (9, 10) and subjected to whole-genome shotgun sequencing.

The 300-bp library (233 to 414 bp) was sequenced using Illumina HiSeq 2000, generating 82,200,000 past-filtered 100-bp paired-end reads, with a coverage of 500×. Reads were *de novo* assembled with Velvet 1/1/06, SOAPdenovo 0.9.5, and GapCloser 1.10 (11–13) (without filtering) generating 448 segments (kmer = 83) using reads with a minimum quality of 30 for each base. The assembly that contains 76 scaffolds of ≥200 bp, covers 5,294,657 bp, with an *N*_50_ of 543,331 bp and a longest segment of 1,472,236 bp and second longest segment of 635,056 bp. Mapping was done using BWA 0.9.5 with a maximum of two differences from the reference sequence per paired-end read and a maximum of one gap not in the 5 bp of the read. A total of 97.96% of the single reads were mapped to the assembly (with 0.37% singletons), and 68% of the single reads were mapped to the *V. vulnificus* YJ016 genome (14). There is evidence for the presence of a plasmid related to pYJ016 (scaffold_27 and contig_C1443).

Various bioinformatics approaches were applied with the aim of reducing the number of contigs without any success, maybe due to the genome complexity of CladeA-yb158(BT1), derived from the high rates of horizontal gene transfer in the *Vibrio* species (5, 6, 15) and the presence of multiple repetitive regions. However, there are three scaffolds that account for more than 50% of the assembly (L50), indicating the high quality of the assembly taking into consideration the genome complexity of this phylogroup.

The draft genome of CladeA-yb158(BT1) consists of 76 segments covering 2 chromosomes and a plasmid (5.29 Mbp; 46.7% G+C content). A total of 4,574 coding sequences (CDS), 82 pseudo genes, 7 rRNAs, 94 tRNAs, and 1 non-coding RNA (ncRNA) were predicted and annotated by the NCBI Prokaryotic Genome Annotation Pipeline (16), similar to the annotation predicted by RAST (17).

Genome comparison of the CladeA-yb158 genome to two published *V. vulnificus* biotype I genomes using the SEED viewer in RAST (17, 18) revealed 88.32% (2,847 I chromosomes, 1,473 II chromosomes, and 34 plasmids) and 91.54% (2,685 I chromosomes and 1,448 II chromosomes) common genes to YJ016 (14) and CMCP6 (5, 19), respectively, suggesting higher resemblance to the CMCP6 strain.

Since clade_A is highly clonal, the genome of the CladeA-yb158 strain provides a representation of this phylogroup, contributing to the understanding of the evolution of this human pathogen in the environment.

### Nucleotide sequence accession numbers.

This whole-genome shotgun project has been deposited at DDBJ/EMBL/GenBank under the accession no. LBNN00000000. The version described in this paper is version LBNN01000000.
